# Development of Styrene Maleic Acid Lipid Particles as a Tool for Studies of Phage-Host Interactions

**DOI:** 10.1128/JVI.01559-20

**Published:** 2020-11-09

**Authors:** Patrick A. de Jonge, Dieuwke J. C. Smit Sibinga, Oliver A. Boright, Ana Rita Costa, Franklin L. Nobrega, Stan J. J. Brouns, Bas E. Dutilh

**Affiliations:** a Theoretical Biology and Bioinformatics, Science4Life, Utrecht University, Utrecht, The Netherlands; b Department of Bionanoscience, Kavli Institute of Nanoscience, Delft University of Technology, Delft, The Netherlands; University of California—Irvine

**Keywords:** SMALPs, bacteriophages, outer membrane proteins, phage-host interactions

## Abstract

The infection of a bacterium by a phage starts with attachment to a receptor molecule on the host cell surface by the phage. Since receptor-phage interactions are crucial to successful infections, they are major determinants of phage host range and, by extension, of the broader effects that phages have on bacterial communities. Many receptor molecules, particularly membrane proteins, are difficult to isolate because their stability is supported by their native membrane environments. Styrene maleic acid lipid particles (SMALPs), a recent advance in membrane protein studies, are the result of membrane solubilizations by styrene maleic acid (SMA) copolymer chains. SMALPs thereby allow for isolation of membrane proteins while maintaining their native environment. Here, we explore SMALPs as a tool to isolate and study phage-receptor interactions. We show that SMALPs produced from taxonomically distant bacterial membranes allow for receptor-specific decrease of viable phage counts of several model phages that span the three largest phage families. After characterizing the effects of incubation time and SMALP concentration on the activity of three distinct phages, we present evidence that the interaction between two model phages and SMALPs is specific to bacterial species and the phage receptor molecule. These interactions additionally lead to DNA ejection by nearly all particles at high phage titers. We conclude that SMALPs are a potentially highly useful tool for phage-host interaction studies.

**IMPORTANCE** Bacteriophages (viruses that infect bacteria or phages) impact every microbial community. All phage infections start with the binding of the viral particle to a specific receptor molecule on the host cell surface. Due to its importance in phage infections, this first step is of interest to many phage-related research and applications. However, many phage receptors are difficult to isolate. Styrene maleic acid lipid particles (SMALPs) are a recently developed approach to isolate membrane proteins in their native environment. In this study, we explore SMALPs as a tool to study phage-receptor interactions. We find that different phage species bind to SMALPs, while maintaining specificity to their receptor. We then characterize the time and concentration dependence of phage-SMALP interactions and furthermore show that they lead to genome ejection by the phage. The results presented here show that SMALPs are a useful tool for future studies of phage-receptor interactions.

## Abstract (precis)

Bacteriophages (viruses that infect bacteria or phages) impact every microbial community. All phage infections start with the binding of the viral particle to a specific receptor molecule on the host cell surface. Due to its importance in phage infections, this first step is of interest to many phage-related research and applications. However, many phage receptors are difficult to isolate. Styrene maleic acid lipid particles (SMALPs) are a recently developed approach to isolate membrane proteins in their native environment. In this study, we explore SMALPs as a tool to study phage-receptor interactions. We find that different phage species bind to SMALPs, while maintaining specificity to their receptor. We then characterize the time and concentration dependence of phage-SMALP interactions and furthermore show that they lead to genome ejection by the phage. The results presented here show that SMALPs are a useful tool for future studies of phage-receptor interactions.

## INTRODUCTION

The first stage of infection by a bacteriophage consists of the viral particle binding to a cognate receptor on the host cell surface (1, 2). Each phage attaches to a specific receptor molecule and, as a result, largely determine the specificity to hosts that is common among characterized phages (3, 4). Indeed, the specificity of phages to their receptors is a useful tool for characterizing bacterial strains (5). In addition to phage applications, the molecules involved in phage-receptor interactions are often evolutionary hot spots due to their crucial role in successful infections (6, 7). As such, understanding phage-receptor interactions is important for understanding the evolutionary dynamics that govern networks of phages and hosts (8) and the role that phages play in regulating bacterial communities (9–11). Indeed, phage-receptor binding is a widely investigated research theme, and studies of phage-receptor interactions have among others ranged from the mechanics of phage binding and DNA ejection (12–14) to the structural modeling of bound receptor molecules (15, 16).

Known phage receptors include a wide variety of diverse molecules on the bacterial cell surface (2, 17). While some phages bind to pili, flagella, or cell capsules, many phage receptors are associated with the bacterial cell envelope (17). Which molecules serve as receptor molecules largely depends on the diversity of bacterial cell wall structures (18). Known receptors in Gram-positive bacteria are mostly present in the peptidoglycan layer (2, 18), while those in Gram-negative bacteria include sugar moieties in lipopolysaccharide (LPS) chains and membrane-incorporated porin and transport proteins (2, 17). Due to their association with the cell membrane, phage receptors, especially membrane proteins, can be challenging to study. Proteinaceous phage receptors are generally incorporated into the bacterial cell membrane and are thereby dependent on this membrane to maintain correct folding (19), which makes them notoriously difficult to isolate (20, 21). This limits the number of phages of which the interaction with a receptor can be studied in detail.

**FIG 1:**
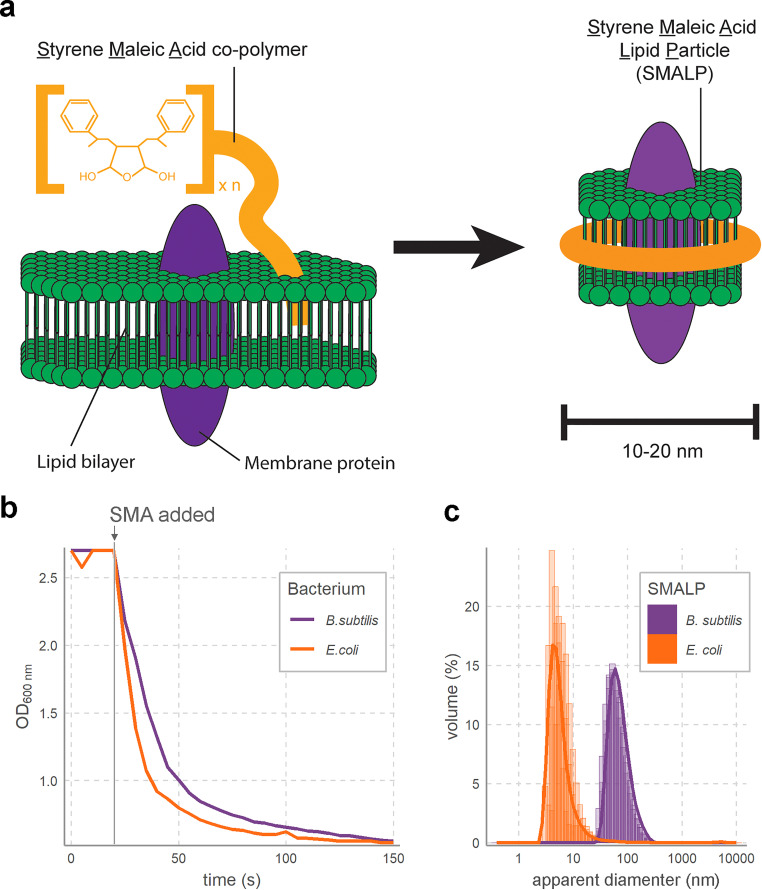
Styrene maleic acid lipid particles (SMALPs) can be made from distinct microbial membranes. (a) Schematic of the process by which styrene maleic acid copolymer incorporates itself into lipid membrane to isolate membrane proteins in SMALPS. (b) SMALPs can be made from Gram-positive and Gram-negative bacterial membranes at roughly equal efficiency. The ODs of E. coli K-12 and B. subtilis 110 NA membrane suspensions (10 mg/ml) rapidly decreased upon addition of 20 mg/ml SMA (at gray vertical line) due to dissolution of large membrane particles into SMALPs. (c) SMALPs of B. subtilis are larger than SMALPs of E. coli, as shown by the diameter distributions of SMALPs from the two species obtained through dynamic light scattering. Each sample was measured in triplicate, with the replicates plotted on top of each other. Solid lines indicate the averages of the three replicates.

Among novel solutions to facilitate membrane protein studies is styrene maleic acid (SMA) (22). SMA is an amphipathic copolymer composed of chains with alternating hydrophobic styrene and hydrophilic maleic acid groups (23). Upon addition of SMA to lipid bilayers, it incorporates itself into the membrane, which leads to the solubilization of membranes into styrene maleic acid lipid particles (SMALPs) (Fig. 1a) (22). In SMALPs, the SMA polymer is wound around a disk of membrane about 10 nm in diameter, with hydrophobic styrene groups intercalated between lipid tail acyl groups and hydrophilic maleic acid groups pointed outward (23). Membrane proteins may be captured within the confines of SMALP discs, and SMALPs thus allow isolation of bacterial membrane proteins while maintaining their natural lipid environment. Since their initial development for membrane protein studies, SMALPs have been used to obtain three dimensional structures of membrane proteins (24, 25), and obtain their biophysical characteristics (22, 26). Because SMALPs are useful agents for bacterial membrane isolation, they are also potentially useful for phage-receptor studies.

In this study, we report on the exploration of SMALPs as a platform to study phage-host interactions. We test whether SMALPs can inhibit phages that infect taxonomically diverse bacteria by binding to diverse membrane-associated receptor molecules. We then show that phages interacting with SMALPs maintain specificity to their receptors and that they eject their DNA after binding to SMALPs.

## RESULTS AND DISCUSSION

### SMALP formation in two taxonomically distant bacteria.

While SMALPs have been developed as a platform to study membrane proteins for over a decade (27), this has mostly focused on Escherichia coli membranes. It is consequently unknown whether differences in membrane composition and cell wall structure between bacterial lineages (28) will lead to differences in membrane solubilization by SMALPs. To test this, we started by solubilizing membranes of two taxonomically distant bacteria, one Gram positive and one Gram negative, with styrene maleic acid copolymer. As sample bacteria, we selected E. coli and Bacillus subtilis. Membrane solubilization of these bacteria, evidenced by a diminishing absorbance at 600 nm upon SMA addition, showed similar rates of SMALP formation in the two bacterial membranes (Fig. 1b). Dynamic light scattering (DLS) revealed that the Gram-negative E. coli membranes formed uniform particles of roughly 10 nm in diameter, as is normally observed for SMALPs (Fig. 1c) (22). Interestingly, DLS of Gram-positive B. subtilis SMALPs showed larger particles of 50 to 100 nm (Fig. 1c). Previous studies reported that SMALP size is dependent on the ratio between polar and apolar groups in the SMA polymer (29) and the ratio between polymer and membrane (30, 31). While no extant studies report on the effects of membrane composition from different bacteria on SMALP size, the distinct lipid composition of E. coli and B. subtilis membranes (32) may lead to differences in SMALP sizes as we observed. Beyond lipid composition, the 40-nm-thick outer peptidoglycan layer (33) that is attached to the outer membrane of B. subtilis by lipoteichoic acids makes cell envelopes much thicker than those of E. coli (34). Since DLS measurements assume a spherical object, this could further contribute to the larger apparent diameter of B. subtilis SMALP.

### SMALPs decrease viable phage counts.

Next, we tested whether SMALPs from both Gram-positive B. subtilis and Gram-negative E. coli bacteria inhibit the lytic activity of three model phages (Table 1). As model phages, we selected one member from each of the three major International Committee on the Taxonomy of Viruses-recognized families of tailed phages (*Myo*-, *Podo*-, and *Siphoviridae*) (35, 36), as the distinct morphologies of these families differentiates their binding mechanisms (1). The three model phages, *Podoviridae Bacillus* phage φ29, *Siphoviridae Escherichia* phage λ and *Myoviridae Escherichia* phage T4, each bind to distinct receptor molecules. *Bacillus* phage φ29, like many phages infecting Gram-positive bacteria (18), binds to cell wall teichoic acids. *Escherichia* phage λ exclusively binds to a maltose porin protein LamB, while *Escherichia* phage T4 binds to both outer membrane protein C (OmpC) and glucose moieties in LPS chains. While some phages bind to other structures than our model phages, most notably pili and flagella (1), the use of SMALPs to study phage-host interactions will evidently have to focus on membrane-associated structures. Our model phages additionally allowed us to test whether, in addition to proteins, LPS and peptidoglycan layers attached to SMALPs are available to phages.

**TABLE 1:** Characteristics of model phages used to test the inhibitory effect of SMALPs

Phage	Family	Host used	Classification	Receptor(s) (reference)
λ	*Siphoviridae*	E. coli BW25113	Gram negative	LamB protein (66)
T4	*Myoviridae*	E. coli BW25113	Gram negative	OmpC protein/LPS (67)
ϕ29	*Podoviridae*	B. subtilis 110 NA	Gram positive	Teichoic acids (37)

**FIG 2:**
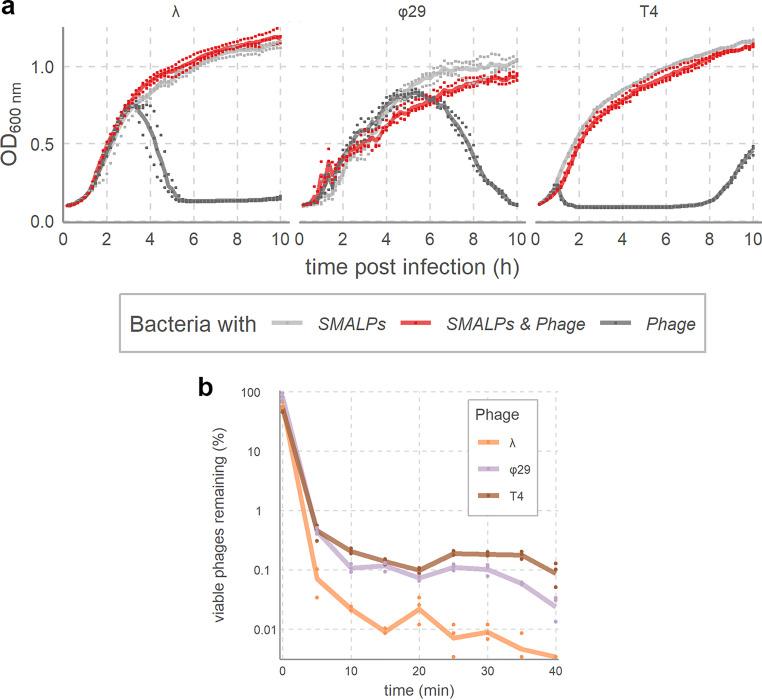
SMALPs decrease viable phage counts. (a) Inhibition of lytic activity by *Bacillus* phage φ29 (10^6^ PFU/ml, starting MOI of 10^−2^) and *Escherichia* phages λ (10^5^ PFU/ml, starting MOI of 10**^−^**^3^) and T4 (10^4^ PFU/ml, starting MOI of 10**^−^**^4^) by SMALPs prepared from their hosts. Liquid cultures of host cells were either not infected (SMALP-only control, light gray), infected with SMALP-incubated phage (red), or infected with untreated phage (phage-only control, dark gray), followed by measurement of the OD_600_ every 10 min. Points are measurements in each biological triplicate; solid lines indicate the mean. (b) Time-dependent decrease of viable phage counts by SMALPs. Samples were taken at 5-min time intervals of phages incubated with SMALPs from their host and used for plaque assays. The percentages of viable phages were estimated using a nontreated phage control. Points indicate replicates, while solid lines indicate means.

As preliminary examination of phage binding by SMALPs, we tested phage lytic capability in liquid cultures after treatment with SMALPs prepared from 10 mg/ml bacterial membrane. Addition of SMALPs to liquid bacterial cultures infected with phage at titers of 10^4^ to 10^6^ PFU/ml phage (starting multiplicity of infection: 10^−4^ to 10^−2^; Fig. 2a) resulted in complete inhibition of phage lytic activity. Differences in the titer at which we observed complete inhibition of phage lysis suggested varying sensitivities to SMALPs among the three phages. Because these phages differ greatly in infection dynamics (e.g., burst sizes from 100 to 1,000) (37–39) and because liquid assays are nonquantitative (40), we next quantified SMALP-phage interactions through plaque counts after SMALP treatment. Viable phage counts at different time intervals revealed that 1 to 0.1% of 10^6^ PFU/ml solutions remained after 5 min of incubation with SMALPs (Fig. 2b). Longer incubations showed a lower rate of decrease in viable phage populations, especially for T4 and φ29, while after 20 min, decreases in viable phage counts largely plateaued. SMALP-phage interactions thus seem to proceed in a two-stage process, with a rapid initial phase followed by a slowed secondary phase, similar to the two-stage adsorption dynamics that are characteristic of phages binding to live host cells (41).

In addition to time-dependent dynamics, we measured the decrease in viable phages upon addition of different concentrations of SMALPs (see Fig. S1 in the supplemental material). Undiluted SMALP stocks used in these experiments had been prepared from 10 mg/ml membrane suspensions and 20 mg/ml SMA, but since some of these materials are removed in the SMALP production process, these values do not accurately represent SMALP concentrations. Hence, in the following experiments, a dilution factor was used instead of concentration counts. Experiments with increasingly diluted SMALPs confirmed that a positive relationship existed between SMALP concentration and phage inhibition. Of the three model phages, φ29 was most sensitive to SMALPs, since 10,000-fold-diluted SMALP stocks still inhibited about 90% of a 10^6^ PFU/ml phage φ29 solution. Since φ29 binds to teichoic acids on the B. subtilis cell surface, its sensitivity to SMALPs indicates that the lipoteichoic acid linkages between membrane and peptidoglycan layers (42) are maintained in the B. subtilis SMALP solutions. Phages λ and T4 exhibited lower sensitivity to SMALPs than φ29, with SMALP dilutions of a 1,000-fold or more having little effect on phage populations. While at high SMALP dilutions, the behavior of these phages is similar, at low dilutions T4 is about 2 orders of magnitude less sensitive than λ. This likely reflects their disparate binding dynamics, since λ engages in a single interaction with a LamB protein (43), whereas T4 requires up to four independent interactions of tail fibers to receptors for successful infection (44). It is likely that each T4 phage binds to a separate SMALP particle. This difference in the number of receptor interactions per phage particle suggests that T4 would need to bind 4-fold more SMALPs to yield a similar decrease in the number of viable phages. Equal SMALP amounts will therefore bind more λ than T4 particles, leading to lower inhibition for T4 than for λ. Meanwhile, the fact that φ29 exhibits the highest sensitivity to SMALPs implies that teichoic acids are present in SMALPs. Teichoic acids are present in larger numbers in the cell wall than protein receptors such as LamB and OmpC, to which λ and T4 bind. This higher abundance of φ29 receptors increases the number of binding events for φ29 and thus decrease of viable phage counts. Together, these results show that for three distinct phages that infect two taxonomically distant hosts, SMALPs are a potential platform for the study of phage receptor.

### SMALPs cause receptor-specific genome ejection by phages.

**FIG 3:**
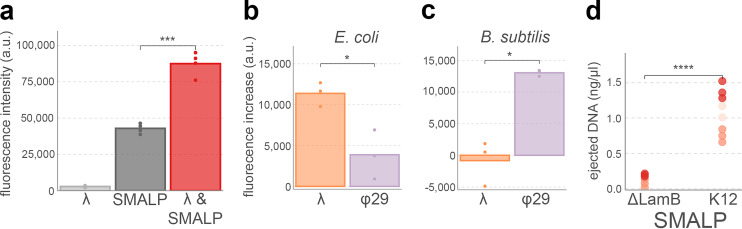
Specificity of ejection by various phages upon addition to SMALPs. (a) Ejection of phage λ DNA in the presence of E. coli K-12 SMALPs. YO-PRO fluorophore was added to phage λ stock (light gray), E. coli K-12 SMALPs (dark gray), and a combination of the two and fluorescence was measured after a 20-min incubation. Points indicate biological replicates. (b and c) YO-PRO fluorescence assays as in panel a were performed with phages λ and φ29 added to E. coli K-12 and B. subtilis SMALPs. SMALP-only measurements were subtracted from the SMALP-plus-phage measurements, phage measurements were negligible (see panel a). (d) Receptor-specific DNA ejection by phage λ after incubation with SMALPs from E. coli K-12 or ΔLamB, as measured by QuBit DNA quantification assays. The experiments were repeated three times, each instance (which have different shades of red) composed of biological triplicates (individual points). SMALP-only measurements were subtracted from SMALP-plus-phage incubations. Significance values were determined using Welch two-tailed *t* tests (*, *P* < 0.05; **, *P* < 0.01; ***, *P* < 0.001; ****, *P* < 0.0001).

To test whether phage-SMALP interactions led to DNA ejection, we measured fluorescence of DNA-specific fluorophores before and after incubation with SMALPs. This experiment employed the YO-PRO DNA stain, which was earlier shown to bind free DNA at a much faster rate than encapsulated phage DNA (45). In addition, this dye has been used to study DNA ejection in *Salmonella* phage P22 (46). We first tested our experimental setup by comparing fluorescence signal of λ phage stocks, E. coli SMALPs, and a combination of the two (Fig. 3a). This revealed that SMALP stocks have sizeable fluorescence intensity when added to YO-PRO DNA stain. Fluorescence measurements of separate polymer confirmed that SMA itself interacts with the DNA stain. This nonspecific interaction with DNA stain likely results from the strong overall negative charge of the SMA polymer (47), making it similar to DNA. Despite this high background fluorescence signal, when we incubated SMALPs with phages, fluorescence significantly increased (two-tailed *t* test, *P* = 0.0007; Fig. 3a). Since the fluorescence of phage stocks was negligible, the increase evidently results from an increase of free DNA. Similar methods with the same DNA stain previously showed that fluorescence increase in *Salmonella* phage P22 in the presence of purified LPS is due to an increase in free DNA after being ejected by phage particles (11). We therefore concluded that phage λ ejected its DNA in the presence of E. coli SMALPs.

Next, to examine whether the phage DNA ejection was specific to SMALPs prepared using the appropriate host, we incubated phages λ and φ29 with SMALPs prepared from either E. coli or B. subtilis. After subtracting background signal from SMALP-only and phage-only samples, phage λ added to E. coli SMALPs had a significantly higher fluorescent signal than phage φ29 added to the same SMALPs (two-tailed *t* test, *P* = 0.03; Fig. 3b). Incubation of the two phages with SMALPs prepared from B. subtilis resulted in the reverse, with significantly higher background-adjusted fluorescence for phage φ29 (two-tailed *t* test, *P* = 0.02; Fig. 3c). From these results, we concluded that both phage λ and φ29 ejected their genomes in the presence of SMALPs prepared from their cognate hosts. In addition, we concluded that SMALP-phage interactions reflect specific phage-host interactions, at least at this large phylogenetic distance (E. coli and B. subtilis are from different bacterial phyla and have different Gram stains).

We further determined the extent of specificity by comparing phage λ DNA ejection when added to SMALPs prepared from E. coli K-12 and E. coli ΔLamB. For these tests, we employed a Qubit fluorometer, which is a calibrated system for DNA quantification. In three repeats of experiments that were each composed of biological triplicates, background-adjusted DNA measures were consistently higher when we added phage λ to E. coli K-12 SMALPs than to E. coli ΔLamB SMALPs (two-tailed *t* test, *P* = 6 × 10^−6^; Fig. 3d). Assuming that background-adjusted values were accurate representations of free DNA amounts, we estimated the fraction of phages which had ejected their DNA. Based on the stock phage titer of 1 × 10^10^ PFU/ml and a molecular weight of 1.5 × 10^7^ g/mol for λ DNA, the theoretical maximum of ejected DNA in our reaction was 0.80 ng/μl. The calibrated measurements identified 1.09 ± 0.28 ng/μl DNA in the reactions. Assuming a slight underestimation (i.e., well within an order of magnitude) of the added phage stock, these results indicated that (nearly) all phage particles ejected their genome. The SMALP-phage interaction is thus highly efficient at prompting DNA ejection in phage λ. Combined, these fluorescence-based experiments provided ample evidence that DNA ejection in SMALP-phage interactions is receptor specific. SMALPs could therefore be a powerful tool in studying phage-receptor interactions, especially for phages that interact with membrane proteins.

### SMALPs as tool to study phage-host interactions.

**FIG 4:**
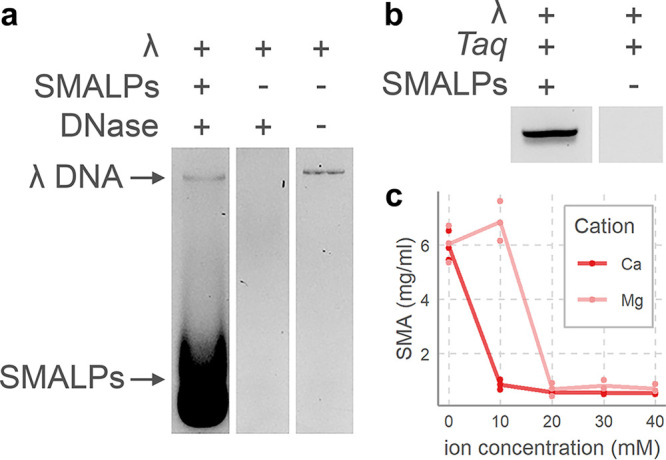
Chelating activity of SMALPs causes inhibition of DNA-modifying reactions. (a) DNase inhibition by SMALPs, as shown by an agarose gel with λ DNA with SMALPs and DNase, λ DNA with DNase, and λ DNA alone. (b) Inhibition of *Taq* polymerase by SMALPs after PCR targeting λ DNA. (c) Precipitation of SMA by addition of divalent cations in calcium chloride (Ca) and magnesium sulfate (Mg), followed by centrifugation. SMA concentration was determined from the adsorption at 259 nm and by comparison to a standard curve (see Fig. S2 in the supplemental material). Points indicate replicates; solid lines indicate averages.

While the above results show SMALPs are useful tools to study phage-host interactions, there are also potential drawbacks to consider. For example, SMALPs are strong chelators of divalent cations (22). This may be particularly problematic in phage-host studies, as some phages are dependent on Mg^2+^ or other divalent cations for successful infection initiation (48, 49). Due to their chelating activity, proteins that are active against DNA, like polymerases, nucleases, or restriction enzymes, may not be active in the presence of SMALPs. Indeed, when we tested the activity of DNase in the presence of SMALPs, we observed inhibition of nuclease activity (Fig. 4a). This also supported the above findings that SMALPs bind to DNA-specific fluorophores, as they form thick smears at low molecular weight when subjected to agarose gel electrophoresis. Similarly, amplification reactions using *Taq* polymerase were entirely inhibited by SMALPs (Fig. 4b). To circumvent this, we developed a method of removing SMALPs from solution. This method is based on their chelating activity, which leads to SMALP precipitation (22). Addition of calcium or magnesium to SMALP solutions above 10 mM successfully removed almost all polymer from solution (Fig. 4c). However, SMALP removal in this fashion could in turn inhibit polymerases, as high concentrations of divalent cations are known to do (50, 51). Calcium particularly inhibits polymerases by outcompeting magnesium as cofactor (51), although high magnesium concentrations also inhibit polymerases (50, 52). In addition, DNA molecules also interact with divalent cations (53), which should be taken into account for further studies of DNA released by phages in the presence of SMALPs. Applications of SMALPs to study phage-host interactions needs further development of alternative means of SMALP removal. Alternatively, several modified SMA polymers that are more positively charged and therefore potentially weaker chelators have recently been developed (54–56). Their adoption for SMALP-phage studies may lift the above-described drawbacks.

### Conclusions.

In this study, we report on the potential utility of SMALPs for studies of bacteriophage-receptor interactions. We found that SMALPs are viable tools, because their interactions with model phage particles result in host- and receptor-specific ejection of phage genomic material. As a result of our findings, SMALPs may see adaptation as a platform to study various aspects of phage-receptor interactions. SMALPs may be useful tool for cryo-electron microscopy of phages binding mechanisms (15, 57–59), since this is implausible when using whole cells due to their size (60). The differences in phage binding to 10-nm-diameter SMALPs and to large bacteria, across the surface of which phages often engage in 2D diffusion (1), might further reveal details of the mechanics by which phages find their receptors. In addition, affinity purification or pulldown assays of phage bound SMALPs coupled with mass spectrometry approaches may aid in receptor identification. While there are drawbacks associated with SMALPs (i.e., their chelation of magnesium), further developments of SMALPs as tools to study bacterial membrane proteins may aid in their future applications in studying phage-receptor interactions.

## MATERIALS AND METHODS

### Bacterial and bacteriophage strains.

To test whether bacterial membranes from different bacteria could be solubilized by the SMA polymer, we used E. coli K-12 BW25113 (DSMZ 27469) and Bacillus subtilis 110 NA (DSMZ 5547). To test specificity of phages to SMALPs, we further used E. coli strains JW3996-1 (ΔLamB), JW3596 (ΔRfaC), and JW2203 (ΔOmpC) from the Keio strain collection (61). The phages used in this study were a virulent mutant of *Escherichia* phage λ obtained from the Westerdijk Fungal Biodiversity Institute (62, 63), *Bacillus* phage φ29 (DSMZ 5546), and *Escherichia* phage T4 (DSMZ 103876). All chemicals were obtained from Sigma-Aldrich, except where stated otherwise.

### Bacterial cultivation and phage production.

Bacteria were cultivated in lysogeny broth (LB) at 37°C under agitation at 180 rpm. The production of phage stocks was according to the soft-agar overlay method, as described previously (64). Phage stocks were kept at 4°C until further use. To enumerate phages, a 0.1-ml exponentially growing bacterial culture was added to 5 ml of 0.7% (wt/vol) LB agarose (Bio-Rad), which was subsequently layered on a 1.5% (wt/vol) LB agar plate. After the top layer had dried, 10 μl of 10-fold dilution ranges of phage stocks in SM buffer (100 mM NaCl, 8 mM MgSO_4_⋅H_2_O, 50 mM Tris-HCl [pH 7.5]) were pipetted onto the agar. The plates were placed at a 45° angle and until the phage dilutions had dried and then incubated overnight at 37°C).

### Styrene maleic anhydride copolymer hydrolyzation.

Styrene maleic anhydride copolymer (SManh) at a 67:33 ratio (Polyscience) was hydrolyzed to styrene maleic acid copolymer as described previously (22). In short, 12.5 g of SManh was suspended in a round-bottom flask containing 250 ml of 1 M NaOH. This flask was connected to a reflux setup, and the suspension was heated in sunflower oil at 98°C for 4 h under constant stirring. Afterward, the suspension was cooled to room temperature, and 6 M HCl was added to a final concentration of 1.1 M to precipitate the hydrolyzed polymer. Styrene maleic acid (SMA) was pelleted by centrifugation at 7,000 × *g* for 20 min; the supernatant was then discarded. The pellet was resuspended in 250 ml of 100 mM HCl and centrifuged at 7,000 × *g* for 20 min; the supernatant was then discarded. This was repeated twice, once with 250 ml of 100 mM HCl and once with deionized water. The SMA pellet was frozen at −80°C and extensively freeze-dried. SMA was dissolved at a concentration of 60 mg/ml in 20 mM Tris-HCl (pH 8) and stored at −20°C until further use.

### Bacterial membrane isolation and SMALP production.

To isolate bacterial cell membranes, bacteria were first grown overnight at 37°C while shaking. Cells were pelleted by centrifugation at 10,000 × *g* and 4°C for 15 min; the supernatant was then discarded. Pellets were washed with the original volume of lysis buffer (50 mM Tris HCl [pH 7.5], 2 mM MgCl_2_), after which the centrifugation was repeated. After the supernatant was discarded, the cells were resuspended in 4 volumes of lysis buffer per g of wet cell weight, to which 1 tablet of cOmplete EDTA-free protease inhibitor was added. To lyse the cells, the suspension was three times passing through a model CF1 cell disruptor (Constant Systems) at 1.5 × 10^7^ Pa. The cell debris was pelleted by centrifugation at 12,000 × *g* and 4°C for 15 min and collecting the supernatant. Next, the membranes were pelleted by centrifuging the supernatants at 225,000 × *g* and 4°C for 1 h. Pellets were resuspended in lysis buffer and centrifuged again. Soluble proteins were removed by resuspending the pellet in 200 mM NaCl plus 20 mM Tris HCl (pH 7.5) and repeating centrifugation. The pellets were dissolved in 20 mM Tris HCl (pH 7.5)–200 mM NaCl at a concentration of 10 mg/ml and stored at −20°C until further use.

To produce SMALPs from bacterial membranes, we suspended membranes in 200 mM NaCl–20 mM Tris HCl (pH 7.5) and added SMA to obtain a final solution with 10 mg/ml membrane and 20 mg/ml SMA (i.e., for a membrane/polymer ratio of 1:2). To allow SMALP formation, solutions were incubated under constant rotation for 20 min at room temperature. Nonsolubilized membrane material and excess SMA were removed by filtration through a sterile 20-μm filter and extensive dialyzing against 20 mM Tris HCl (pH 7.5). SMALP solutions were stored at 4°C until further use.

The effects of divalent cations on the SMA polymer were examined using CaCl_2_ and MgSO_4_. To 14 mg/ml SMA polymer, 0 to 30 mM CaCl_2_ or MgSO_4_ was added, and the optical density at 259 nm (OD_259_) was measured in a cuvette using a NanoPhotometer C40 (Implen). The concentration of SMA in every sample was calculated using a linear standard curve ranging from 0 to 40 mg/ml SMA.

To determine SMALP sizes, we used dynamic light scattering using a Zetasizer ZS instrument (Malvern), using default software settings and multiple narrow modes analysis of the correlation data. Before measurement, samples were briefly degassed and equilibrated for 300 s at room temperature.

To test the efficacy of SMALP dissolution for different bacterial membranes, membrane suspensions of 10 mg/ml were prepared in 200 mM NaCl–20 mM Tris HCl (pH 7.5) in 1.5-ml cuvettes. Subsequently, SMA was added to a final concentration of 20 mg/ml, and the OD_600_ was monitored over time in a NanoPhotometer C40, with measurements every 10 s.

### Effect of SMALPs on phage lytic activity.

Equal volumes of SMALP solution and phage stock at 10-fold dilutions between 10^1^ and 10^10^ PFU/ml were mixed, followed by incubation for 20 min at room temperature to allow phages to bind SMALPs. SMALP and phage dilutions were prepared in sterile dilution buffer (100 mM NaCl plus 50 mM Tris HCl [pH 7.5]) where necessary. Bacteria were grown to an OD_600_ of 0.5, and 10 μl of bacterial suspension was diluted in 89 μl of fresh LB medium in a 96-well plate. Diluted bacteria were incubated at 37°C under agitation for 30 min, and then 1 μl of SMALP/phage suspension was added. To bacterium-only controls, 1 μl of dilution buffer was added instead. The 96-well plate was then incubated in a Synergy H1 microplate reader (BioTek) at 37°C under continuous double orbital shaking for 10 h, during which the OD_600_ was determined every 10 min.

### Characterizing SMALP-phage interaction with agar plate assays.

To determine the effect of incubation length on SMALP-phage interactions, equal volumes of phage (at 10^6^ PFU/ml) and SMALP solutions were mixed. At 5-min time intervals between 0 and 40 min, 20-μl aliquots were retrieved, to which CaCl_2_ was added to a final concentration of 20 mM. As a negative control to establish original viable phage counts, phage stock at 10^6^ PFU/ml was diluted in an equal volume of 200 mM NaCl plus 20 mM Tris HCl (pH 7.5), to which CaCl_2_ was added to a final concentration of 20 mM. After a 2-min centrifugation at 21,000 × *g*, the supernatant was retrieved and used to enumerate viable phage particles as described under “Bacterial cultivation and phage production.” To test the effect of SMALP concentration on phage inhibition, 10-fold SMALP dilutions were made in 100 mM NaCl plus 50 mM Tris HCl (pH 7.5). Samples were subsequently prepared and enumerated as described for the time trials, except that only incubation times of 20 min were used. Negative controls were produced as described for the time trials. Decreases in viable phage particles were calculated by dividing phage titers after the reaction by those obtained from negative controls. For both time trial and SMALP concentration experiments, all samples consisted of biological triplicates.

### Spot assays to determine specificity.

SMALP and phage reactions, as well as negative controls were performed as described in the previous section, with incubation times of 20 min. After incubation, 10-μl spots of reaction mixture were placed on top of a double-layer agar plate prepared as described under “Bacterial cultivation and phage production.” Spots were dried at room temperature and subsequently incubated for 16 h at 37°C.

### Transmission electron microscopy.

Before transmission electron microscopy, phage stocks were purified using a preformed CsCl density gradient. A two-step gradient was prepared in thin-walled ultracentrifuge tubes (Beckman Coulter) using CsCl at densities of 1.6 and 1.4 g/ml. On top of these layers, 1 ml of a 10^11^ phage preparation was placed, and density gradients were centrifuged at 111,000 × *g* for 2 h using a Sw60Ti swinging-bucket rotor (Beckman Coulter). After centrifugation, white phage layers were collected by puncturing the tubes with a hypodermic needle, as described previously (65). To remove excess CsCl, phages were cleaned by three consecutive washes with SM buffer in an Amicon 30-kDa spin filter (Merck), which was centrifuged for 10 min at 3,000 × *g*. Samples with SMALPs were then prepared as described under “Characterizing SMALP-phage interaction with agar plate assays” with an incubation time of 20 min and phage titers of 10^10^ PFU/ml. Samples were applied to thin carbon-coated 400 square mesh copper grids (Electron Microscopy Sciences), which were ionized by glow discharged 90 s. On top of the grids, 3-μl samples were carefully pipetted, followed by incubation at room temperature for 1 min. Liquid was removed using filter paper (Whatman). The grids were then washed three times with 10 μl of Milli-Q water, each time removing the liquid with filter paper. Finally, 3 μl of 2% uranyl acetate was pipetted onto the grids. After a final 30-s incubation at room temperature, the uranyl acetate was removed with filter paper. TEM imaging used a Philips CM200 (200 kV), while micrographs were captured using a TemCam-F416 4 kkD (TVIPS) at 150,000× magnification using EM-MENU software.

### Fluorescence DNA ejection assays.

The first fluorescence assay tested DNA ejection with *Escherichia* phage λ and SMALPs prepared from E. coli K-12 BW25113 membranes. Phage stock was diluted to 10^6^ PFU/ml using SM buffer. Phage stock, SMALP stock, and the two combined were incubated with 1.1 μM YO-PRO-1 iodine (491/509; Invitrogen) for 20 min at 37°C, as described previously for P22 and S. enterica LPS (46). The fluorescence was then measured using a QuBit 4 fluorometer (Thermo Fisher), which used an excitation wavelength of 430 to 495 nm and measured emission at 510 to 580 nm. Samples were composed of biological triplicates. To determine specificity, *Escherichia* phage λ, *Bacillus* phage φ29, E. coli K-12 BW25113 SMALPs, and B. subtilis 110 NA SMALPs were measured separately and in every possible combination of phage and SMALP in the same way.

For further quantification of DNA ejection after SMALP concentration, the assay was repeated with 1 × 10^10^ PFU/ml *Escherichia* phage λ and SMALPs from E. coli K-12 BW25113 and ΔLamB, using a dsDNA HS assay kit and a Qubit fluorometer (Thermo Fisher Scientific). This assay consisted of three repeats, with each repeat consisting of biological triplicates. We calculated the theoretical amount of DNA that was present in the phages in the sample according to the following equation:

$$\text{[DNA]}=\left(\frac{M_{\text{DNA}}}{A}\right)\times {\text{10}}^{\text{6}}\left[\phi \right]\text{,}$$

where [DNA] is the DNA concentration in ng/μl, *M*_DNA_ is the molecular weight of λ DNA (1.5 × 10^7^ PFU/ml), *A* is Avogadro’s number, and ϕ is the phage titer in the reaction (3.2 × 10^10^ PFU/ml).

### SMALP assays with divalent cations.

To determine the effect of SMALPs on DNase activity, we prepared SMALPs from bacterial membranes as described above. Three samples were made for the assay, the first containing 9 μl of SMALP solution, 10 ng of phage λ DNA (New England Biolabs), and 2 U of DNase I. The second sample replaced the SMALPs with deionized water, and the third sample replaces both the SMALPs and DNase I with water. The samples were incubated at 37°C for 20 min and ran on a 1% (wt/vol) agarose gel for 30 min at 20 V/cm.

To test inhibition of *Taq* polymerase by SMALPs, PCRs were performed containing 1× *Taq* polymerase Master Mix (NEB), 0.2 μM forward (TACGCCGGGATATGTCAAGC) and reverse (TACGCCAGTTGTACGGACAC) primers that target the phage λ E gene, and 0.1 ng of phage λ DNA. In one sample, 10× diluted SMALP solution was added. PCR program consisted of 30 s at 95°C, followed by 25 cycles of 30 s at 95°C, 30 s at 55°C, and 60 s at 72°C, and then finally 5 min at 72°C. Samples were then run on agarose gel as described above.

The effect of the divalent cations Ca^2+^ and Mg^2+^ was tested as follows. SMA stocks of 6 mg/ml were incubated for 20 min in the presence of 0 to 40 mM either CaCl_2_ (Sigma) or MgCl_2_. SMA precipitate was pelleted by centrifuging for 1 min at 21,000 × *g*. Next, the adsorption of the supernatant was determined at 259 nm on a NanoPhotometer C40. Concentrations were calculated using an SMA standard curve with concentrations ranging from 0 to 40 mg/ml.

## Supplementary Material

Supplemental file 1
