# Supplementary material for: Development of Styrene Maleic Acid Lipid Particles as a Tool for Studies of Phage-Host Interactions
Source: J Virol. 2020 Nov 9;94(23):e01559-20. doi: 10.1128/JVI.01559-20 (PMC7654272; doi:10.1128/JVI.01559-20)
Supplement: Supplemental file 1 [file JVI.01559-20-s0001.pdf]

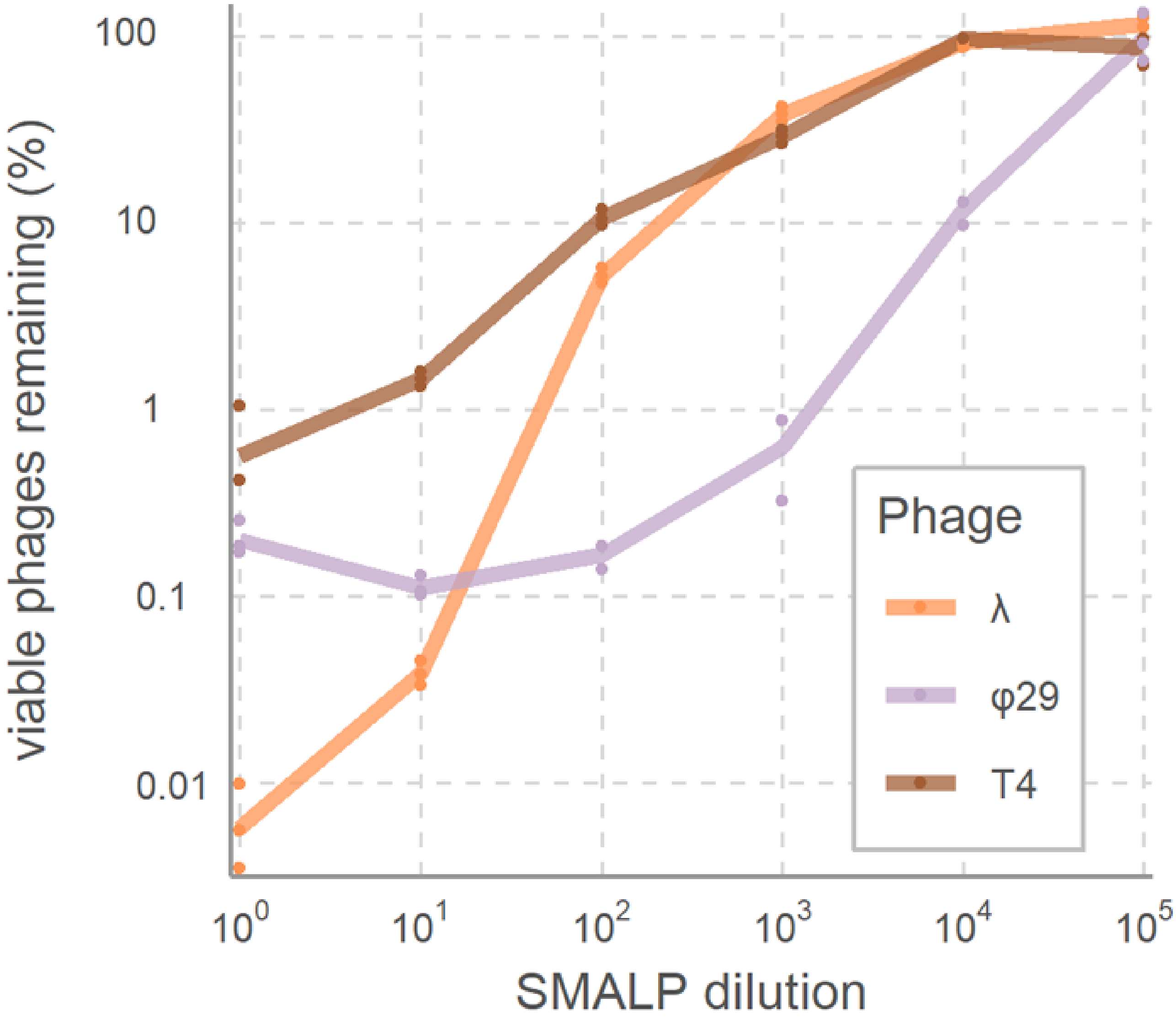

Figure S1: Phage sensitivity to SMALPs shown by treatments with increasingly diluted SMALPs. Experiment was carried out in the same manner as for (b), but with 20-minute time interval and tenfold dilutions of 10 mg/ml (original membrane concentration) SMALP stocks. Both (b) and (c) used 10^6 pfu/ml phage stocks.

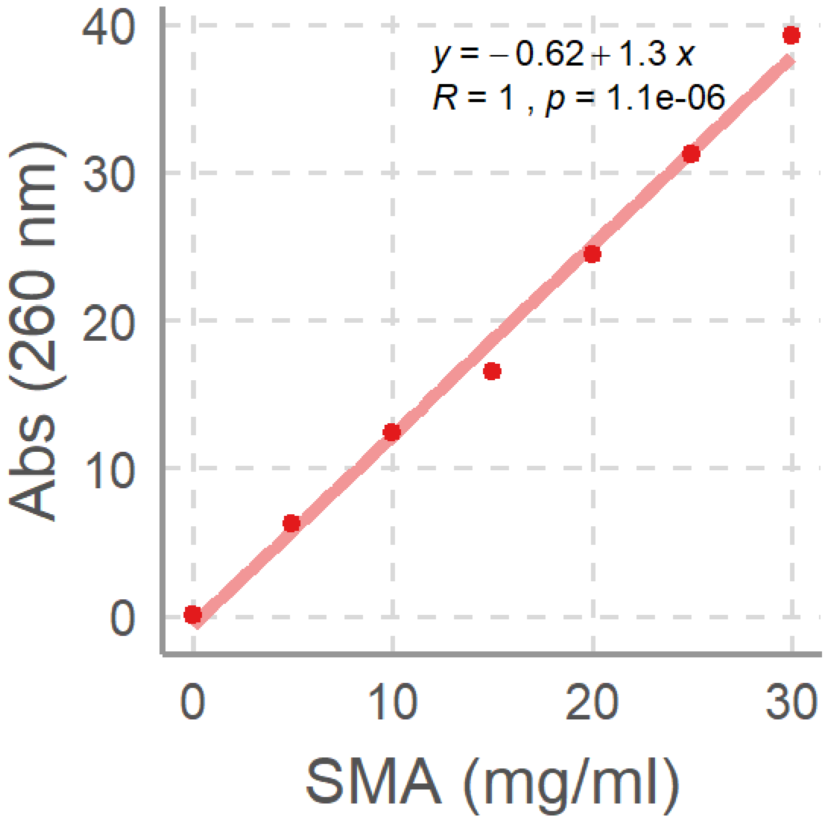

Figure S2: Standard curve used to calculate SMA concentrations in Figure 4c.

Table S1: Raw values belonging to Figure 3d

Phage added	**SMALP**	**Repeat**	**Replicate**			**average background**	**Background-adjusted**		
			1	2	3		1	2	3
TRUE	Wild Type	1	5.040	5.200	5.300		1.007	1.167	1.267
FALSE	Wild Type	1	3.920	3.920	4.260	4.033			
TRUE	ΔLamB	1	3.040	3.320	3.340		-0.140	0.140	0.160
FALSE	ΔLamB	1	3.080	3.140	3.320	3.180			
TRUE	Wild Type	2	1.360	1.180	1.270		0.838	0.658	0.748
FALSE	Wild Type	2	0.658	0.330	0.578	0.522			
TRUE	ΔLamB	2	0.424	0.310	0.368		0.129	0.015	0.073
FALSE	ΔLamB	2	0.416	0.258	0.212	0.295			

TRUE	Wild Type	3	5.180	5.020	4.940		1.520	1.360	1.280
FALSE	Wild Type	3	3.420	3.740	3.820	3.660			
TRUE	ΔLamB	3	2.180	2.200	2.160		0.193	0.213	0.173
FALSE	ΔLamB	3	1.700	2.400	1.860	1.987			
